# Supplementary material for: Effect of Residual Oxygen Concentration on the Lattice Parameters of Aluminum Nitride Powder Prepared via Carbothermal Reduction Nitridation Reaction
Source: Materials (Basel). 2022 Dec 14;15(24):8926. doi: 10.3390/ma15248926 (PMC9787569; doi:10.3390/ma15248926)
Supplement: Supplementary file 1 [file materials-15-08926-s001.zip › materials-2066713-supplementary.pdf]

Supplementary Materials

Effect of Residual Oxygen Concentration on the Lattice Parameters of Aluminum Nitride Powder Prepared via Carbothermal Reduction Nitridation Reaction

Jaegyeom Kim [1,†], Heewon Ahn [1,†], Seung-Joo Kim [2,3], Jong-Young Kim [1,*] and Jae-Hwan Pee [1,*]

[1] Icheon Branch, Korea Institute of Ceramic Engineering and Technology (KICET), 3321, Gyeongchung Rd., Sindun-Myeon, Icheon-si 17303, Republic of Korea
[2] Department of Chemistry, Ajou University, Suwon 16499, Republic of Korea
[3] Department of Energy Systems Research, Ajou University, Suwon 16499, Republic of Korea
* Correspondence: jykim@kicet.re.kr (J.-Y.K.); pee@kicet.re.kr (J.-H.P.); Tel.: +82-31-645-1426 (J.-H.P.)
† These authors contributed equally to this work.

S1. Al Solid-state NMR analysis

^{27}Al Solid-state NMR spectra of AlN raw materials are shown in Figure S1, which evidences that most of residual oxygen is lattice oxygen dissolved in AlN crystals with tetrahedral symmetry. Commercial AlN powder (E-grade, Tokuyama inc., Japan) was used as a reference. In the NMR spectra of our materials (Figure S1), main peaks at 120 ppm due to AlN_4 tetrahedra are observed, and shoulders at ~110 ppm are also found, corresponding to 4-coordinated Al(AlN_3O tetrahedra). As CRN reaction time increases from 2h to 6h for our AlN materials, peak intensity at ~110 ppm, which corresponds to AlN_3O defect, decreases as shown in Figure S1(a).[1,2] Around 10 ppm, no discernible peaks due to 6-coordinated Al defect (AlO_6) were found for our AlN materials (2-6h) as shown in Figure S1(a). The AlO_6 sites are present in grain boundary or surface of AlN grains in a small amount, and therefore XRD peaks due to Al_2O_3 were absent or very weak for our AlN materials. On the other hand, commercial materials exhibits almost no shoulder peak(~110 ppm) compared to our AlN materials and peaks at ~10 ppm, due to AlO_6 defect, which means most of the residual oxygen is not present in AlN grains but grain boundary or second phase. Even though oxygen content of commercial material is almost the same as that of AlN(5 or 6h),(Table 1) however, the oxygen dissolved in AlN grain is much smaller for commercial materials, which is shown by AlN_3O NMR peak area (4.73% and 0% for AlN (5h) and commercial, respectively).

[1] Fitzgerald, J.J.; Kohl, S.D.; Piedra, G.P. Observation of Four-Coordinate Aluminum Oxynitride ($AlO_{4-x}N_x$) Environments in AlON Solids by MAS ^{27}Al NMR at 14 T. *Chem. Mater.* **1994**, 6, 1915-1917.

[2] Jung, W.; Chae, S. ^{27}Al MAS NMR spectroscopic identification of reaction intermediates in the carbothermal reduction and nitridation of alumina. *Mater. Chem. Phys.* **2010**, 123, 610–613.

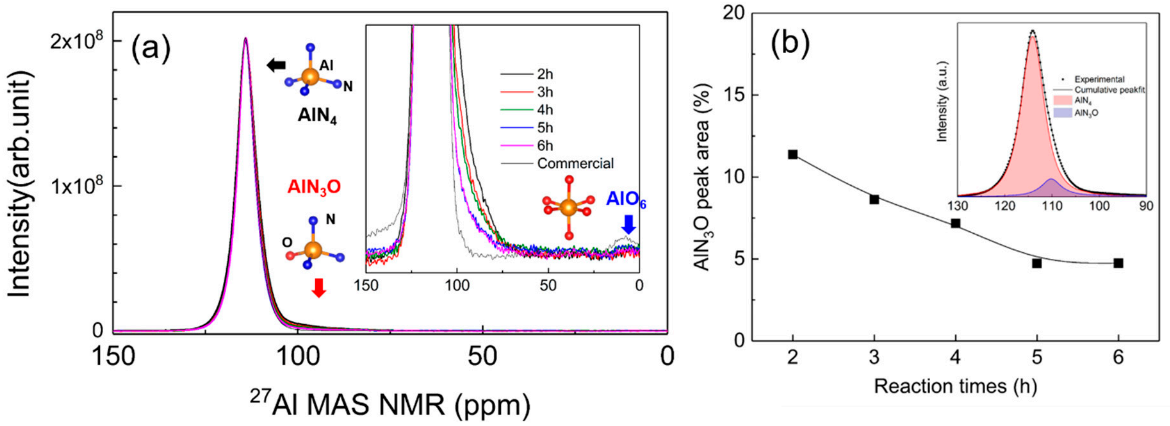

Figure S1. (a) ^{27}Al Solid state NMR spectra for AlN materials showing shoulder peaks (~110 ppm) due to AlN_3O defects. The peaks at ~110 ppm show gradual decrease in intensity with increasing reaction time. Commercial material shows zero intensity in peak due to AlN_3O defect. Very weak peaks due to AlO_6 defect are found for our AlN and commercial materials. (See inset for enlarged image) (b) Fitted peak area due to AlN_3O defect of the main peak is plot as a function of carbothermal reaction time. Fitting of experimental spectra with AlN_3O and AlN_4 profiles is shown in inset.(J. Kim et al. Direct Evidence on Effect of Oxygen Dissolution on Thermal and Electrical Conductivity of AlN Ceramics Using Al Solid-State NMR Analysis. Materials 2022, 15, 8125. https://doi.org/10.3390/ma15228125)

The ^{27}Al solid-state NMR spectra of the sintered AlN samples are shown in Figure S2. In the NMR spectra of the sintered AlN_C-S samples, the peaks (shoulder) at ~110 ppm, excluding the main peak (~120 ppm), a significantly weakened by sintering, which means that the defect due to oxygen substitution in the AlN lattice (i.e., O_N) was removed. Instead, new peaks due to the Al-O defects are found in the spectra of the sintered samples. The new peaks at 0 and 60–70 ppm correspond to the 4-coordinated $AlNO_3$ of a probable γ-AlON phase, whereas the peaks at ~10 ppm correspond to the 6-coordinated AlO_6 cluster, like those in the spectra of the raw materials [1,2]. As the carbothermal reaction time increases from 2 h to 6 h for AlN_C-S materials, peaks due to the γ-AlON phase gradually decrease, whereas peaks at 10 ppm due to the AlO_6 octahedra increase, as shown in Figure S2. As carbothermal time increases, the intensity of the shoulder at ~110 ppm also decreases for the sintered samples, which corresponds to 4-coordinated Al (AlN_3O).

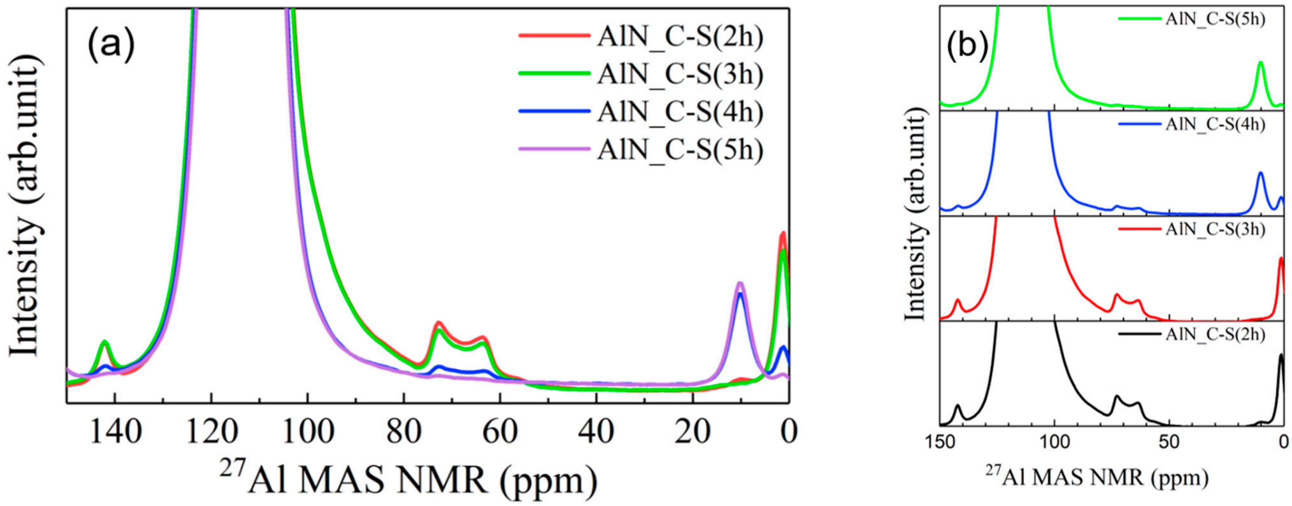

Figure S2. (a) Al solid-state NMR spectra for sintered samples (AlN materials with carbothermal reaction time of 2–5 h). After sintering, the peaks due to AlN_3O defect (~110 ppm) near main peak (AlN_4) were removed, as shown in Table 1. (b) As carbothermal reaction time increases (decreasing oxygen concentration), peaks due to $AlNO_3$ defect of intermediate γ-AlON (60–70 ppm) decrease with increasing peak intensity due to AlO_6 defect (~10 ppm).

S2. HRTEM analysis

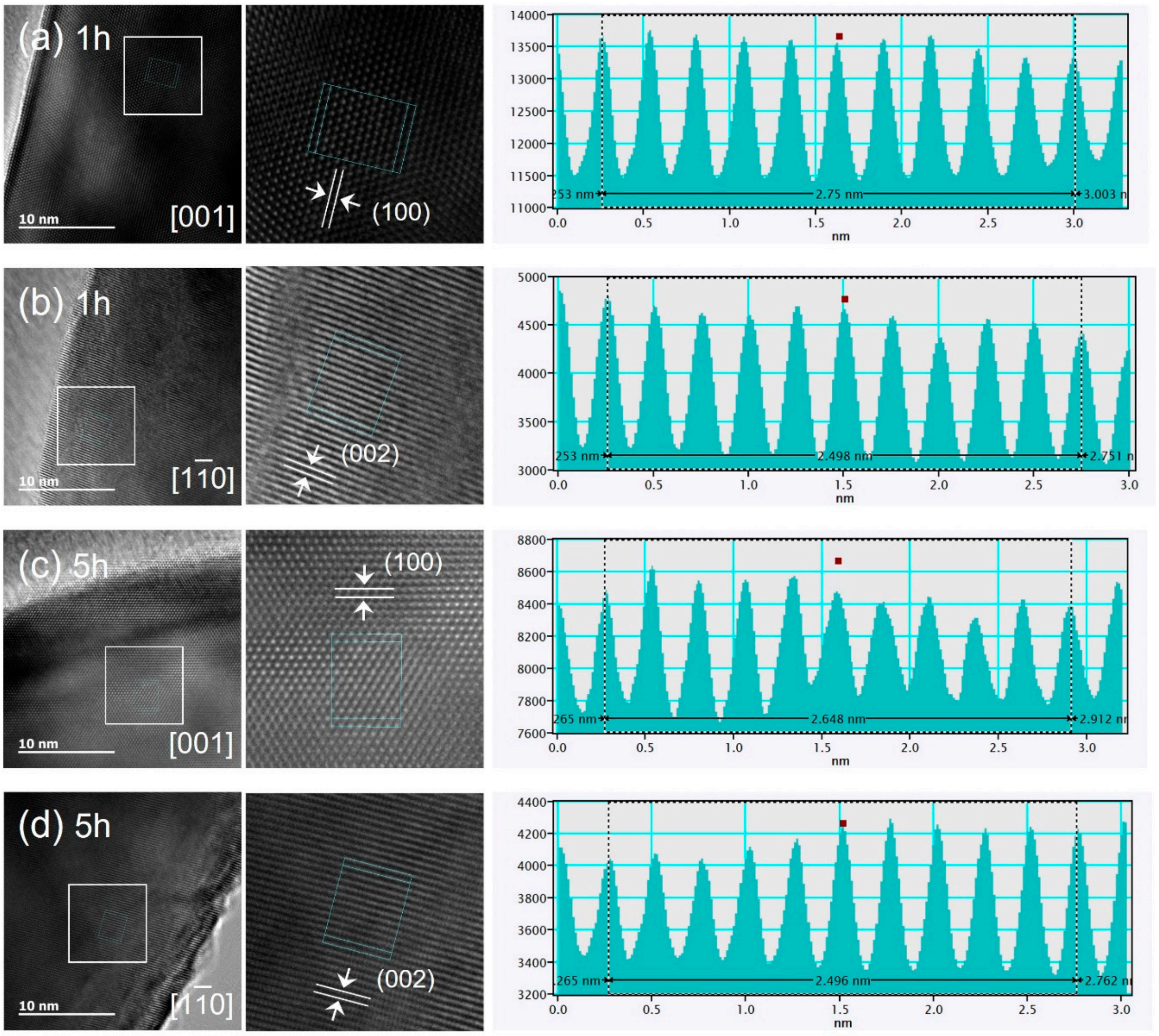

Figure S3. HRTEM images and lattice distance profiles of 1h and 5h samples.

S3. Williamson−Hall analysis

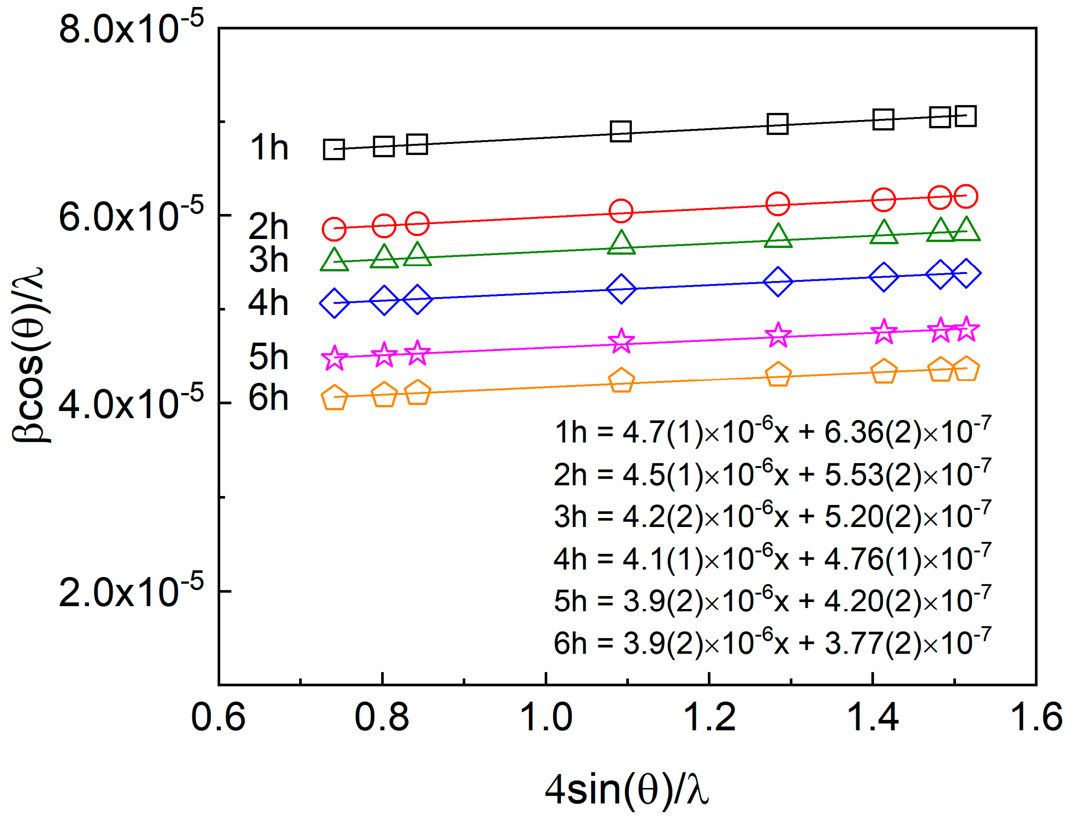

Figure S4. Williamson−Hall plots of AlN samples with different reaction times.
